# When fluoropolymers recycle themselves: self-propagating chemistry for ambient fluoropolymer upcycling

**DOI:** 10.1093/nsr/nwag452

**Published:** 2026-07-22

**Authors:** Alexandru Vlad

**Affiliations:** Division of Molecular Chemistry, Materials and Catalysis, Institute of Condensed Matter and Nanosciences (MOST-IMCN), Université catholique de Louvain, Belgium

Few synthetic polymers embody a greater paradox than polytetrafluoroethylene (PTFE). The exceptional strength of the carbon–fluorine (C–F) bond underpins its outstanding chemical stability, yet the same feature makes fluoropolymers among the most persistent forms of plastic waste. For decades, recycling has largely relied on supplying ever larger amounts of external energy through pyrolysis, mechanochemistry or electrochemical activation to overcome one of chemistry’s strongest single bonds [1]. The challenge is therefore not simply to cleave C–F bonds, but to rethink how the energy required for their activation is delivered.

**Figure 1.**
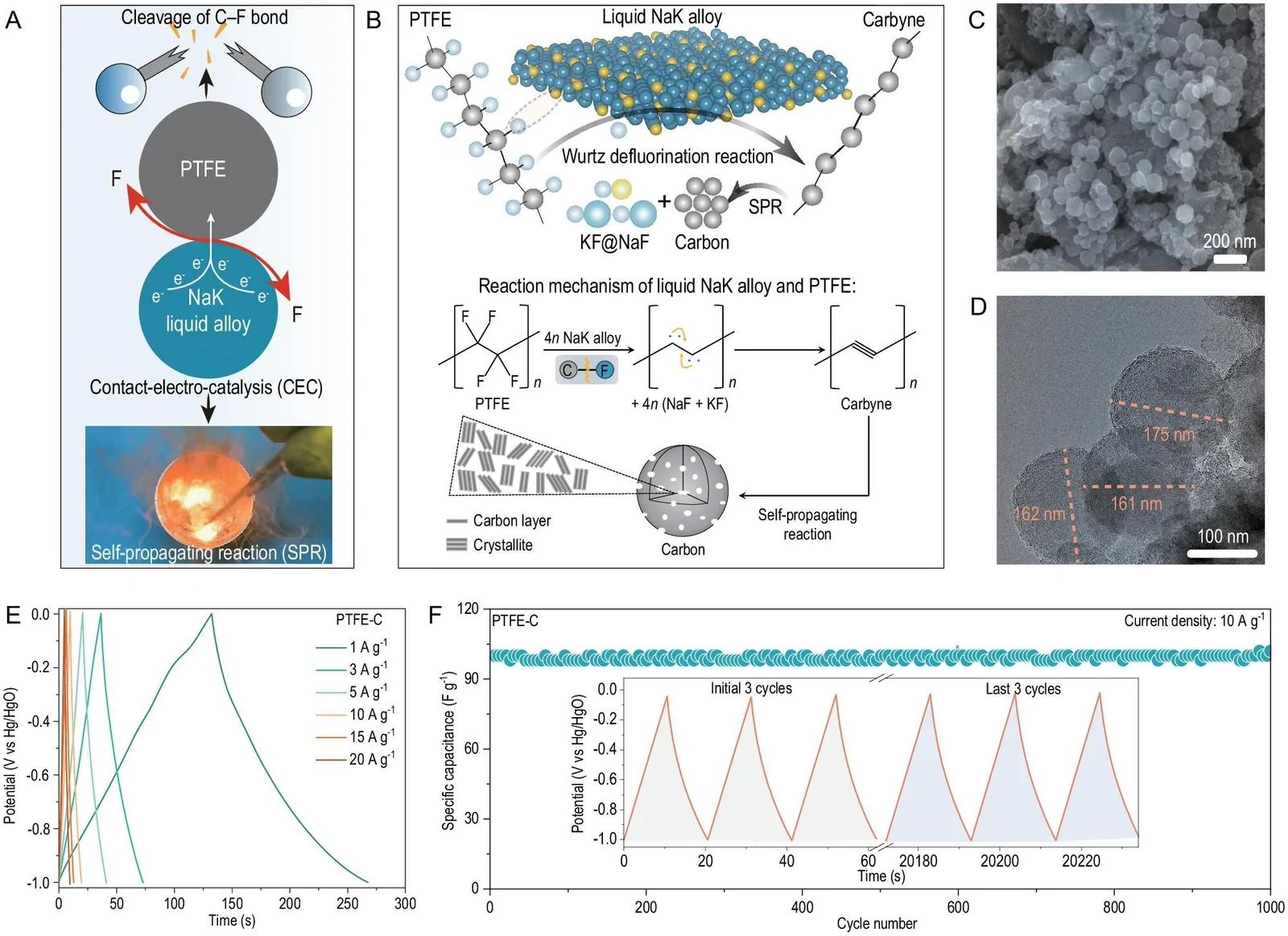
From initiation to self-propagating fluoropolymer upcycling. (A and B) Contact between PTFE and liquid NaK initiates interfacial electron transfer, triggering a self-sustaining Wurtz-type defluorination cascade and carbon reconstruction. (C–F) Representative morphology and proof-of-concept electrochemical performance of the resulting fluorine-doped porous carbon illustrate the value-added products obtained through this chemistry. Reproduced from Ref. [5] with permission.

Recent advances have demonstrated that PTFE is not intrinsically unrecyclable. Mechanochemical activation, photocatalysis and electrochemical approaches have progressively expanded the toolbox for controlled defluorination [2–4]. In a recent study published in *National Science Review*, Liu and co-workers report a conceptually different strategy based on a contact-electro-catalysis-induced self-propagating reaction using a liquid NaK alloy (Fig. 1) [5].

The conceptual advance is not merely the use of a liquid alloy, but the replacement of continuous external energy input by a self-sustaining reaction cascade. Mechanical agitation initiates interfacial electron transfer, triggering a Wurtz-type defluorination process that rapidly becomes self-propagating. Once initiated, the highly exothermic chemistry provides the driving force for continued C–F bond cleavage, fundamentally reframing fluoropolymer recycling from energy-intensive bond breaking to reaction-network design.

The reaction simultaneously converts PTFE into fluorine-doped porous carbon together with alkali-metal fluorides, illustrating how persistent fluoropolymer waste can become a feedstock for functional materials rather than merely being destroyed. The resulting porous carbon shows attractive supercapacitor performance and CO_2_ adsorption capability, demonstrating the practical value of coupling waste remediation with materials upcycling [5].

Perhaps the broader significance of this work lies in the general framework it proposes: initiation, electron-driven defluorination and structural reconstruction. More broadly, it exemplifies an emerging philosophy in sustainable chemistry and green engineering, where persistent waste streams are transformed into valuable products through intelligently designed reaction pathways rather than increasing external energy input [6,7]. If generalizable, such self-propagating chemistries could establish a new design principle for the upcycling of fluoropolymers and other highly inert materials.
